# Monitoring Processes and Their Neuronal Correlates as the Basis of Auditory Verbal Hallucinations in a Non-clinical Sample

**DOI:** 10.3389/fpsyt.2021.644052

**Published:** 2021-10-11

**Authors:** Helena Storchak, Justin Hudak, Thomas Dresler, Florian B. Haeussinger, Andreas J. Fallgatter, Ann-Christine Ehlis

**Affiliations:** ^1^Psychophysiology and Optical Imaging, Department of Psychiatry and Psychotherapy, University Hospital Tübingen, Tübingen, Germany; ^2^Center on Mindfulness and Integrative Health Intervention Development, College of Social Work, University of Utah, Salt Lake City, UT, United States; ^3^LEAD Graduate School & Research Network, University of Tübingen, Tübingen, Germany

**Keywords:** inner speech, auditory verbal imagery, fNIRS, fMRI, auditory verbal hallucinations (AVH)

## Abstract

Auditory verbal hallucinations (AVH) are a characteristic symptom of psychosis. An influential cognitive model accounting for the mechanisms in the generation of AVHs describes a defective monitoring of inner speech, leading to the misidentification of internally generated thoughts as externally generated events. In this study, we utilized an inner speech paradigm during a simultaneous measurement with functional near-infrared spectroscopy (fNIRS) and functional magnetic resonance imaging (fMRI), in order to replicate the findings of neural correlates of inner speech and auditory verbal imagery (AVI) in healthy subjects, reported in earlier studies, and to provide the first validation of the paradigm for fNIRS measurements. To this end, 20 healthy subjects were required to generate and silently recite first and second person sentences in their own voice (inner speech) and imagine the same sentences in a different, alien voice (AVI). Furthermore, questionnaires were deployed to assess the predisposition to acoustic hallucinations and schizotypal traits to investigate their connection to activation patterns associated with inner speech and monitoring processes. The results showed that both methods, fNIRS and fMRI, exhibited congruent activations in key brain areas, claimed to be associated with monitoring processes, indicating that the paradigm seems to be applicable using fNIRS alone. Furthermore, the results showed similar brain areas activated during inner speech and monitoring processes to those from earlier studies. However, our results indicate that the activations were dependent more on the sentence form and less on the imaging condition, showing more active brain areas associated with second person sentences. Integration of the sentence construction into the model of inner speech and deficient monitoring processes as the basis for the formation of AVHs should be considered in further studies. Furthermore, negative correlations between questionnaires' scores and activations in precentral gyrus and premotor cortex indicate a relationship of schizotypal characteristics and a deficient activation pattern.

## Introduction

Schizophrenia is a major mental disorder affecting ~1% of the general population. It describes a heterogeneous group of illnesses with dysfunctions in brain structure, chemistry and function, manifesting in a heterogeneous clinical presentation and course of disease. Positive symptoms, which constitute a distortion of normal psychological functions occurring in acute psychotic states (e.g., hallucinations, delusions, and thought disorder), are one of the main clinical features of schizophrenia (e.g., hallucinations, delusions, and thought disorder) (1–3). Auditory verbal hallucinations (AVH) affect 60–80% of patients with schizophrenia (4). They are defined as auditory perceptions (i.e., most typically verbal, in the form of voices) without an external stimulus, usually assuming a derogative and distressing content (5). The underlying pathological mechanisms are still not clearly understood, but a range of theories have been proposed. An influential cognitive model accounting for the mechanisms in the generation of AVHs describes a defective monitoring of inner speech, leading to the misidentification of internally generated thoughts and images as externally generated events (6, 7). Inner speech constitutes a mental thinking in words (8), which allows us to communicate with ourselves by “developing an auditive-articulatory image of speech, without uttering a sound” (9) (p.391). According to the stated theory, inner speech is not perceived as self-generated but is misinterpreted as an external signal due to defective monitoring processes (7). The theory is based on an internal forward model for sensorimotor integration, which makes predictions about a performed action (e.g., arm movement) by comparing the current state with the motor command (10). According to this model, which was put forward by Sperry and by von Holst and Mittelstaedt in 1950 [adapted from (11)], an *efference copy*, described as corollary discharge (12), is created in parallel to the representation of the motor command (13) and is used to predict the future state and corresponding sensory feedback. Discharges from the frontal lobe “prime” the auditory cortex that self-generated actions (or speech) is about to be produced (14). The prediction of the desired state is available (approx. 50–100 ms) before the actual motor action is performed (15), making the individuals aware of the upcoming movement. If the predicted sensory feedback matches the actual sensory feedback, then they cancel each other out, leading to an attenuation of the perceived sensory information of the motor action. The movement is hence perceived as self-generated. If a mismatch of prediction and feedback occurs, due to defective mechanisms, then there is no compensation of the efference copy and the reafference from the actual sensory feedback, as no sensory attenuation is perceived. Hence, the motor action catches one's attention. This can lead to the impression that the action is externally controlled (16, 17) or “passively experienced as performed by an alien “other”” (17) (p. 393). Feinberg (11) transferred this concept to schizophrenic symptoms and described thoughts as a complex form of motor activity and the process as being similar to internal feedback and corollary discharge in motor acts. He assumed that disturbances of the feedback processes might be linked to psychopathological symptoms of schizophrenia and considered them to cause deficits in determining the origin of thoughts, whether they are self-generated or externally controlled, leading to the experience that the thoughts arise independently.

The concept of inner speech as the primary material of AVHs is widely accepted [e.g., (17–20)]. Inner speech and AVH share several common characteristics. Both constitute a form of internal and verbal mental activity, are often related to current events and activities and can comment or regulate the behavior (21). The most common reported neural correlate of inner speech is the left inferior frontal gyrus (IFG), associated with covert and overt speech production (8, 22–24). Shergill et al. (24) found additional brain areas associated with inner speech: the supplementary motor area (SMA), insula, inferior and superior parietal lobe. It is claimed that the monitoring process, rather than the production, of inner speech seems to be deficient in patients with schizophrenia. As the production of inner speech is a process we are used to, it does not need thorough inspection. Studies show that patients with schizophrenia exhibit deficits in the monitoring process during tasks deploying high demands on the monitoring system, e.g., when imagining another person's voice (22, 24), and show fewer differences to healthy subjects while performing tasks with low levels of monitoring, e.g., reciting sentences in their own voice (8, 25). Mental imagery involves several processes: the production and perception of inner speech, retrieval of memory content (26), in the case of a voice, the mental imitation of that voice, as well as the inspection of the process. The mental imagery of speech, also called *auditory verbal imagery (AVI)*, is associated with activation in left IFG, bilateral temporal cortex, SMA, premotor cortex, left precentral and postcentral gyri, inferior parietal lobe, right insula and posterior cerebellar cortex bilaterally (8, 22–24). Results from neuroimaging studies investigating the neural correlates of AVH have shown comparable activated brain regions: IFG, superior temporal gyrus (STG), middle temporal gyrus (MTG) and inferior parietal lobule, anterior insula, precentral gyrus, frontal operculum, and hippocampus/parahippocampal region (bilateral) (27, 28). These findings support the assumption that AVHs might be associated with monitoring processes of inner speech, as identical brain areas are involved. It is hypothesized that one's own inner speech might be misinterpreted as not being self-generated but from an external source; thus, it needs more inspection and activates more brain areas, beyond the ones associated with inner speech.

The aim of this study was to replicate the findings of neural correlates of inner speech and different monitoring processes in healthy subjects and, for the first time, to validate the paradigm for fNIRS measurements, in order to investigate neural correlates of these mechanisms in patients with schizophrenia in a subsequent fNIRS study and potentially link them to AVH. To this end, the inner speech paradigm was utilized during a simultaneous fNIRS-fMRI measurement. The combination of both methods has the advantage of overcoming limitations of a single method. A simultaneous recording with fMRI provides anatomical information about where the fNIRS optodes were located on the individuals' head as well as the underlying brain areas, allowing an accurate spatial assignment, as fNIRS does not provide anatomical data. The advantages of fNIRS include a better temporal resolution of the hemodynamic response measurement, its higher insensitivity to movement artifacts and high external validity, its easy application and high compliance (29), making it easier to apply in patients with schizophrenia.

Furthermore, questionnaires were deployed to assess the predisposition to acoustic hallucinations and schizotypal traits to investigate the connection between the predispositions and activation patterns associated with inner speech and monitoring processes. In this study, an inner speech and imagery paradigm was deployed, where subjects were required to generate and silently recite sentences in a predefined form in their own voice or imagine the same sentences in a different, alien voice. The paradigm was based on earlier studies, which investigated inner speech and monitoring processes. McGuire et al. (8, 22, 23) deployed second person inner speech (own voice) and second person imagery (alien voice) to investigate monitoring processes. Shergill et al. (24) deployed a paradigm with four conditions: first person inner speech (own voice), first person imagery (imagining own voice), second and third person imagery (alien voice). Normal inner speech is thought to be generated in first person (30), whereas AVHs are mostly experienced in second or third person (5). We aimed at examining first and second person sentences to approach this difference. Our paradigm was comprised of four conditions: first person (“I am …”) (1) and second person (“You are…”) inner speech (2) (own voice) and first person (3) and second person (4) imagery (different/alien voice).

We expected that:

- All conditions would elicit activations in the left IFG.
- All AVI conditions would elicit activations in brain areas previously reported to be associated with monitoring of speech: the left IFG, bilateral temporal cortex, SMA, premotor cortex, left precentral and postcentral gyri and inferior parietal lobule.
- Second person inner speech would be associated with more activations than first person inner speech, as, according to study results, inner speech is mainly generated in first person (30), so the generation of second person sentences will probably need more inspection, thus involving more brain areas.
- Second person AVI would be associated with more activations than first person AVI, as the generation of second person sentences will probably put more demands on the monitoring process.

## Materials and Methods

### Participants

A total of 20 healthy subjects [10 female, 10 male; mean age: 28.04 (SD = 4.97)], recruited through a University-based mailing list, participated in this simultaneous fNIRS-fMRI study. All participants were right-handed and had achieved at least a University degree (Bachelor). The participants reported no history of any neurological or mental disorder. This study was approved by the Ethics Committee of the Medical Faculty of the University and the University Hospital of Tübingen (089/2013BO2) and was conducted in accordance with the latest version of the Declaration of Helsinki. Participants provided written informed consent and were compensated with 30 Euros for participating in the study.

### Procedure

On the measurement day, we collected anamnestic data, the participants filled out the questionnaires (see section Questionnaires) and the combined fNIRS-fMRI measurement was conducted, comprising an anatomical measurement (7 min), followed by a functional measurement while the first part of the paradigm was performed (15 min), then a resting-state measurement (7 min), the second part of the paradigm (15 min) and an emotional paradigm (7 min), which will not be part of the current report. The measurement lasted for about 50 min with an additional preparation time of about 30 min.

### Questionnaires

All participants completed the *Mehrfach-Wortschatz-Intelligenztest*, Version B (MWT-B) (31), to assess general intelligence, the *Launey Slade Hallucination scale* [LSHS-R; (32)] to assess the predisposition to hallucinatory experiences, the *Varieties of Inner Speech Questionnaire* (VISQ) (33) for acquisition of phenomenological characteristics of inner speech, the German version of the *Schizotypal Personality Questionnaire* (SPQ-G) (34) to assess schizotypal personality traits and the *Oxford Liverpool Inventory of Feelings and Experiences* (O-LIFE) (35), a self-rating questionnaire for schizotypal symptom assessment. We translated the items of the English versions of the questionnaires into German and an English native speaker translated it back into English to validate the translated questionnaires. The questionnaires were deployed to assess the predisposition to acoustic hallucinations and schizotypal traits to investigate the connection between these and activation patterns associated with inner speech and monitoring processes.

### Inner Speech Paradigm

During the simultaneous fNIRS-fMRI measurement, an inner speech paradigm a modified version of the inner speech task by McGuire et al. (8, 23) was used to elicit neural activation associated with the production, processing and monitoring of inner speech. The task was programmed in Presentation version 22 (Neuro Behavioral Systems, United States). The paradigm was split into two identical parts, one part with first person sentences and the other part with second person sentences. The order of the two tasks was randomized and a resting-state measurement of 7 min (which will not be part of the analysis) was conducted between them to minimize habituation effects. The paradigm was composed of three different conditions. For all conditions, adjectives from an item pool of 120 adjectives, comprised of complimentary (e.g., successful), derogatory (e.g., boring) and neutral (e.g., awake) words, were presented on the screen. The neutral words were compiled and rated in regard to their neutral interpretation for a previous pilot study, to investigate the feasibility of the current paradigm. The complimentary and derogatory adjectives derived from the item pool used in the study of McGuire et al. (8, 23).

In the *control condition*, the participants had to read the adjectives, which were presented on the screen, silently in their minds and without moving their lips [*reading words (RW)* condition]. In total, 15 neutral adjectives in 3 blocks of 5 words were shown, each word for 4 s with an interstimulus interval (ISI) of 1 s. A fixation cross was presented between each block for 14 s. In the other two conditions, 45 adjectives per condition were presented in 9 blocks of 5 words (same presentation duration and ISI), comprising 3 blocks with neutral adjectives, 3 blocks with derogatory adjectives, and 3 blocks with complimentary adjectives, in a randomized order. In the *inner speech (INS)* condition, the participants had to build first person sentences with the adjectives presented on the screen (“I am …+ adjective,” e.g., “I am clever.”) and to recite them in their own inner speech (silently, in their minds, without moving their lips). The *monitoring [auditory verbal imagery (AVI)]* condition, where the monitoring of inner speech was operationalized, was identically constructed, but the sentences had to be *imagined* in a different, alien voice (The voice was not predefined, each participant had to imagine a voice which would be alien to her/him). Before each condition, an instruction with the sentence form was shown on the screen for 5 s to indicate the next condition. The form of the sentences with derogatory, complimentary and neutral adjectives was chosen to imitate the form of AVHs (e.g., “I am stupid,” “I am intelligent.”, “I am awake.”), as most of the patients with schizophrenia experience AVHs in this or a similar form (5). Furthermore, the pre-defined form, where only the last word of the sentence differed (the adjective presented on screen) and the first part of the sentence was fixed (and not seen), should ensure that the generation of the sentences was easier for the participants and they could concentrate more on the imagining of the sentences. The three conditions were presented in a randomized order. The paradigm lasted for 15 min. The second paradigm part was identically constructed, but with second *person* sentences, which had to be built and recited (e.g., “You are clever.”).

### Data Acquisition

#### fNIRS Acquisition

fNIRS and fMRI were recorded simultaneously. We used a continuous-wave, multi-channel fNIRS system (ETG-4000 Optical Topography System; Hitachi Medical Co., Japan) to measure the relative concentration changes in oxygenated (O_2_Hb) and deoxygenated (HHb) hemoglobin (relative to a pre-recorded baseline) at a sampling rate of 10 Hz. The MRI-compatible probe-set consisted of 22 channels arranged in a 3 × 5 optode array, containing 8 emitters and 7 detectors with a fixed emitter-detector distance of 30 mm. The probe-set was oriented according to the 10–20 system for EEG electrodes placement (36) and covered fronto-temporo-parietal areas on the left side of the head. For the probe-set placement we used the data we obtained from a neurofeedback study [see (37) for the anatomic channel assignment using a neuronavigation system (LOCALITE GmbH, St. Augustin, Germany)]. The fNIRS probe-set on the head was covered with a cap to fixate the optodes. Furthermore, cushions were used to fixate the head in order to minimize head movement artifacts. The fNIRS system was placed outside the scanner in a separate room, as it was not MRI-compatible. To connect the probe-set underneath the MRI-head coil to the ETG-4000 we used MRI-compatible 10-m optic fibers, passing a cable tunnel in the wall.

#### fMRI Acquisition

The structural and functional MRI measurements were conducted on a 3 T Siemens MAGNETOM Prisma MRI scanner (Siemens, Erlangen, Germany). We used a 12-channel head coil, as this was big enough for the fNIRS probe-set to fit in. The structural images (T1-weighted) were recorded using a 3D magnetization prepared rapid gradient echo (MPRAGE) sequence with a voxel size of 1 × 1 × 1 mm, a repetition time (TR) of 2,300 ms and an echo time (TE) of 3.05 ms. The functional imaging was performed with gradient echo planer imaging (EPI) sequences, with a TR of 2,000 ms and TE of 30.03 ms [80° flip angle, 52 slices, 2.5 mm thickness, field of view (FOV) 210 × 210 mm, 84 × 84 matrix, 2.5 × 2.5 mm in-plane resolution]. The fMRI and fNIRS time series were synchronized by the sixth EPI volume, which triggered the start of the functional task.

### Analysis

#### fNIRS Analysis

For the offline fNIRS data analysis, custom scripts were programmed in MATLAB (MATLAB R2017; The MathWorks, Natick, MA, USA). The analysis was performed for each paradigm separately (first person and second person sentences). The raw data was pre-processed applying the following steps: a bandpass filter (0.01–0.3 Hz) to remove physiological artifacts, the correlation-based signal improvement algorithm from Cui et al. (38) and a wavelet-based transform (39) were used to correct for motion artifacts (detection threshold: 1.5 SD above the range of the data) (40) and interpolation of manually-inspected channels was employed to correct for channels with poor signal to noise ratio using a Gaussian distribution, where proximal channels were weighted higher than distal channels. Because of the supine position of the participants, the upper optodes of the probe-set were partially elevated from the head surface resulting in noisier channels mainly in the parietal lobe and thus were more likely to be interpolated. Using triggers, the data was separated into 7 blocks: *RW*; *INS*: neutral adjectives, positive adjectives, negative adjectives; *AVI:* neutral adjectives, positive adjectives, negative adjectives. For each block, the average amplitude across the 30 s block was calculated with a 5-s baseline correction. For statistical analysis, 5–25 s of each block (mean value) were used per condition and participant.

The fNIRS channels were assigned to the underlying cortical brain areas using anatomical information from the MPRAGE sequence (see section Anatomical Assignment). Based on prior studies investigating inner speech, we defined regions of interest (ROIs) by averaging the amplitudes of the included channels (see Table 1). Furthermore, the single conditions were summarized to *INS* and *AVI*, as we did not expect differences in activations between the different adjectives. The amplitudes of the single ROIs were extracted. As the paradigm was conducted separately (one part with first person sentences and the other part with second person sentences) and we did not have an explicit hypothesis for a direct comparison of the different sentence forms, but the different conditions (INS vs. AVI), separate analyses of variance (ANOVAs) were conducted for every ROI, first with the conditions INS, AVI and baseline, to investigate a general activation pattern associated with the paradigm, and subsequently with RW, INS and AVI, to investigate the neural correlates of inner speech and differing monitoring processes, using IBM SPSS Statistics 22 (Armouk, NY, USA). For significant main effects, *post-hoc* two-tailed Student's *t*-tests were performed (with Bonferroni-Holm correction for multiple comparisons). In cases where the assumption of normality was violated (tested for normal distribution using Kolmogorov-Smirnov test), we used two-tailed Mann-Whitney *U*-tests.

**Table 1:** ROIs with assigned fNIRS channels (all on the left hemisphere).

**Brain areas (ROIs)**	**Channels**
IFG	4, 8, 13
Premotor cortex	12, 21
Precentral gyrus	16
Postcentral gyrus	11, 20
Wernicke's area	6, 10
Supramarginal gyrus	15, 19
Angular gyrus	14
MTG	2

Furthermore, the amplitudes of the ROIs in the conditions INS and AVI were contrasted against the amplitudes of the RW condition, separately for each sentence form, and the resulting amplitudes were correlated with the scores of the questionnaires to investigate the relationship between the predisposition to acoustic hallucinations and schizotypal traits and the activation pattern associated with different monitoring processes.

#### fMRI Analysis

The fMRI analysis was performed for each paradigm separately (first person and second person sentences). The first five EPI volumes were discarded to account for magnetization saturation effects. The raw data was pre-processed using Statistical Parametric Mapping software (SPM) 12 applying a slice-time correction, a motion correction and spatial normalization. For the motion correction, we conducted a realignment using the time series of each subject with the respective mean EPI image as a reference. The functional scans were coregistered with each anatomical scan. We conducted an automatic anatomical segmentation with the structural images to divide the data into its different components—background, scalp, skull, cerebrospinal fluid, gray matter and white matter voxels. The images were spatially smoothed with an 8 mm full-width at half maximum (FWHM) Gaussian smoothing kernel and a high-pass filter of 1/128 Hz was applied on the time series of each voxel. We conducted a model-based regression analysis where the time series of each subject were modeled voxel-wise for each condition [*RW, INS* (neutral, positive and negative adjectives), AVI (neutral, positive and negative adjectives)] with additional regressors for motion parameters from the pre-processing. Parameters (β-weights) of each regressor were estimated using the general linear model. The conditions INS and AVI were contrasted separately against RW. In the second-level group analysis these contrasts were tested against zero using *t*-tests. To determine regions of activation, the threshold was set to *p* = 0.05 (whole brain, uncorrected) with a minimum voxel size of 10. Furthermore, the amplitudes of the regions of activation in the conditions INS and AVI (contrasted against the RW condition) were extracted, separately for each sentence form, and, using IBM SPSS Statistics 22 (Armouk, NY, USA), correlated with the scores of the questionnaires to investigate the relationship between the predisposition to acoustic hallucinations and schizotypal traits and the activation pattern associated with different monitoring processes. To compare the results of the correlations with the fNIRS data, coefficients of determination (R^2^) were calculated to examine which method explained more variance in the (sub) clinical scales.

#### Anatomical Assignment

To identify the exact spatial optode positions, we used the segmented structural MRI images. The optodes were visible as indentations on the skin, so that the coordinates for each channel and each subject were identified, extracted and normalized to MNI space (using Statistical Parametric Mapping software SPM 12). The channel positions were averaged across subjects and the coordinates were projected on a brain template with a probabilistic assignment of fNIRS channels to Brodmann Areas based on automatic anatomical labeling (AAL) (41–43) (for further information).

## Results

### Questionnaires and Task Ratings

All subjects reported that they were able to perform the task during the measurement, with a higher reported difficulty in the AVI condition (*m* = 4.33, SD = 1.89) than in the INS condition (*m* = 2.46, SD = 2.12) (1 = very easy, 10 = very difficult). In the questionnaires assessing the predisposition to schizotypal traits or to hallucinatory experiences, the participants showed scores in a lower range (see Table 2).

**Table 2:** Results in the questionnaires assessing the predisposition to schizotypal traits and hallucinatory experiences.

**Questionnaire**	**n**	**Min**	**Max**	**M**	**SD**	**Range**
LSHS-R	19	1	21	8.4	4.88	0–48
VISQ	20	31	75	46.05	12.05	18–108
SPQ-G	20	0	38	10.6	9.02	0–74
O-Life	20	4	52	21.15	11.20	0–108

*N, sample size; Min, lowest score; Max, highest score; M, mean score; SD, standard deviation*.

### fNIRS Data

**Figure 1:**
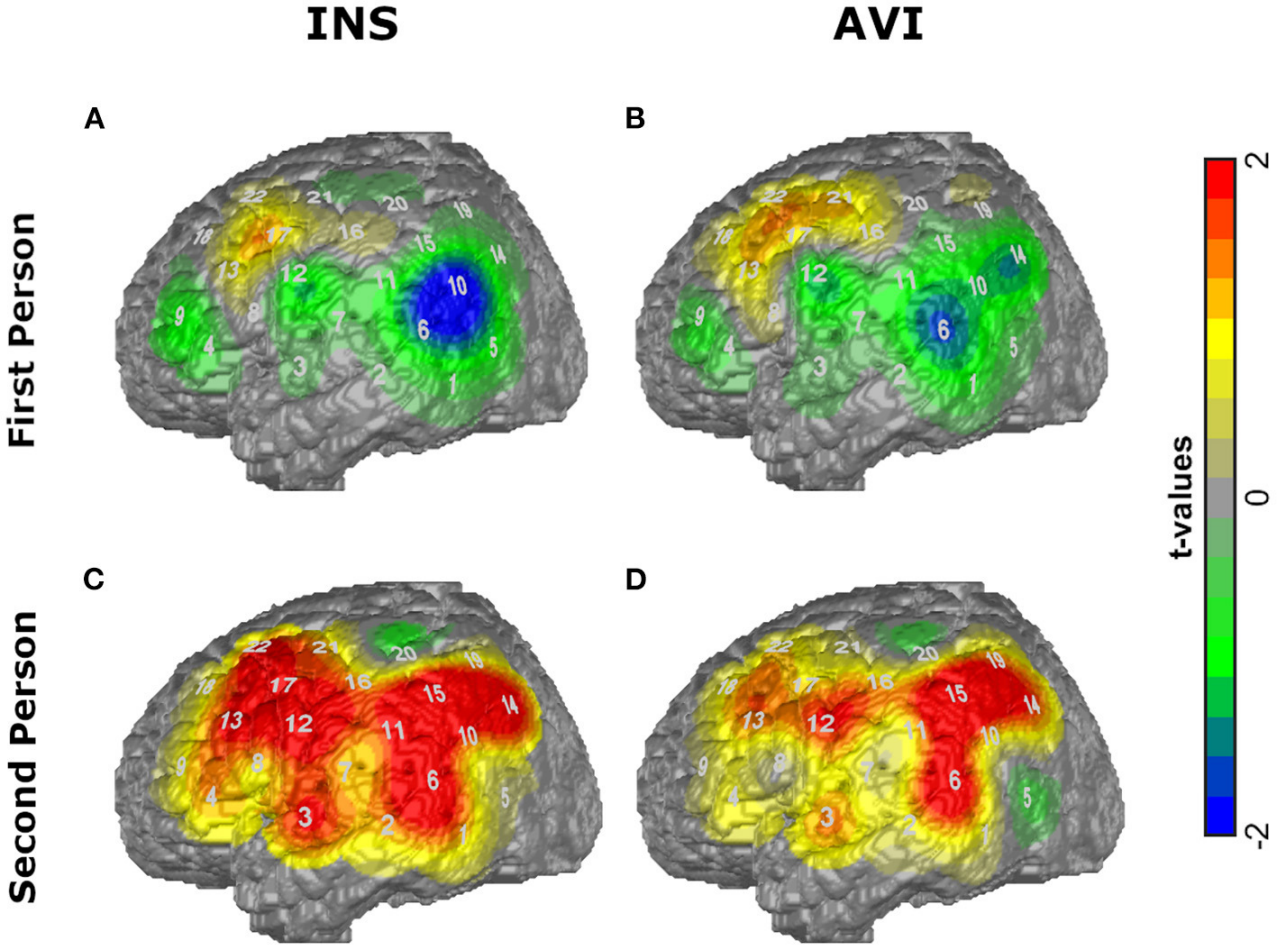
T-maps of the average amplitudes of O_2_Hb for the conditions **(A)** first person INS, **(B)** first person AVI, **(C)** second person INS, **(D)** second person AVI contrasted against RW.

The activation maps in the conditions INS and AVI for both sentence forms (first and second peson) are depicted in Figure 1.

#### First Person Sentences

The ANOVA revealed a significant main effect of condition (including INS, AVI and baseline) for the following ROIs: IFG (*F*_(2, 57)_ = 4.663, *p* = 0.013) and supramarginal gyrus (SMG) (*F*_(2, 57)_ = 4.966, *p* = 0.010). *Post-hoc* Mann-Whitney *U*-test revealed significant differences between *INS* and *baseline* in IFG (*U* = 114, z = −2.33, *p* = 0.02) and SMG (*U* = 105, z = −2.57, *p* = 0.009) and between *AVI* and *baseline* in IFG (*U* = 94, z = −2.87, *p* = 0.004) and SMG (*U* = 104, z = −2.59, *p* = 0.009).

The ANOVA with the conditions INS, AVI and RW did not reach significance.

#### Second Person Sentences

The ANOVA revealed a significant main effect of condition (including INS, AVI and baseline) for the following ROIs: IFG (*F*_(2, 57)_ = 13.382, *p* < 0.001), Wernicke (*F*_(2, 57)_ = 8.360, *p* = 0.001), MTG (*F*_(2, 57)_ = 6.634, *p* = 0.003), premotor cortex (*F*_(2, 57)_ = 6.271, *p* = 0.003) and angular gyrus (*F*_(2, 57)_ = 5.398, *p* = 0.007). *Post-hoc t*-tests and Mann-Whitney *U*-tests revealed significant differences between the conditions *INS* and *Baseline* in IFG (t_(38)_ = 4.384, *p* < 0.001), Wernicke (t_(38)_ = 4.271, *p* < 0.001), MTG (*U* = 73, z = −3.44, *p* = 0.001), premotor area (*U* = 64, z = −3.68, *p* < 0.001) and angular gyrus (t_(38)_ = 3.684, *p* = 0.001) and a significant difference between *AVI* and *baseline* in IFG (t_(38)_ = 3.908, *p* < 0.001), Wernicke (t_(38)_ = 3.394, *p* = 0.002) and MTG (*U* = 102, z = −2.65, *p* = 0.007).

The ANOVA with the conditions INS, AVI and RW revealed a significant main effect of condition for the following ROIs: Wernicke (*F*_(2, 57)_ = 4.025, *p* = 0.023), premotor cortex (*F*_(2, 57)_ = 4.740, *p* = 0.012), SMG (*F*_(2, 57)_ = 7.952, *p* = 0.001) and angular gyrus (*F*_(2, 57)_ = 4.313, *p* = 0.018). *Post-hoc t*-tests revealed significant differences between the conditions *INS* and *RW* in Wernicke (t_(38)_ = 2.68, *p* = 0.011), premotor area (*U* = 112, z = −2.38, *p* = 0.017), SMG (*U* = 108, z = −2.49, *p* = 0.012) and angular gyrus (*U* = 121, z = −2.14, *p* = 0.033) and a significant difference between *AVI* and *RW* in SMG (*U* = 93, z = −2.89, *p* = 0.003).

### fMRI Data

The fMRI analysis revealed significant activations for each condition which are listed in Tables 3.1–4.

**Table 3.1:** Main foci of activation for first person sentences in INS condition contrasted against RW.

**Region**	**Brodman Area (BA)**	**MNI coordinates**	**Cluster size**	**Peak** ** (z score)**
Prefrontal cortex	10	12; 40;−8	10	1.86
Premotor cortex	6	12;−12 64	21	2.13
Primary somatosensory cortex	1	−40;−22; 28	23	2.12
Superior temporal gyrus	22	−44;−38; 16	28	2.67
Anterior cingulate cortex	24	−4; 36;−2	47	2.62
Parahippocampal	30	−30;−50; 6	40	3.07
gyrus	28	−18;−28;−14	11	2.08
Amygdala	–	−20;−8;−12	65	2.99
Putamen	–	−26;−2; 14	18	2.47
		26; 12; 2	10	2.37
Thalamus	–	12;−26; 22	40	3.51
Culmen	–	8;−34;−6	18	2.36
Insula	13	−32; 12;−20	22	2.27

**Table 3.2:** Main foci of activation for first person sentences in AVI condition contrasted against RW.

**Region**	**Brodman Area (BA)**	**MNI coordinates**	**Cluster size**	**Peak** ** (z score)**
Premotor cortex	6	−50; 0; 44	36	2.97
		26;−14; 58	15	2.40
Parahippocampal gyrus	30	−30;−52; 8	67	3.61
Amygdala	–	−20;−8;−10	24	2.63
Putamen	–	−26; 2; 14	21	2.49
Caudate	–	−10;−16; 26	38	2.51
		12;−20; 24	40	2.52

**Table 3.3:** Main foci of activation for second person sentences in INS condition contrasted against RW.

**Region**	**Brodman Area (BA)**	**MNI coordinates**	**Cluster size**	**Peak** ** (z score)**
Prefrontal cortex	8 9 10	26; 8; 36 −30; 14; 42 10; 42; 18 −16; 42; 18 12; 58; 0	96 15 68 126 45	3.19 2.76 2.97 2.63 2.69
Superior temporal gyrus	22	46;−4;−6	51	3.04
Premotor cortex	6	−50;−6; 50	54	2.52
Primary motor cortex	4	−18;−22; 54	64	2.88
Anterior cingulate cortex	24	−4; 38;−2	160	2.66
	32	10; 40; 10	68	2.97
	31	−20;−40; 46	74	2.78
Fusiform gyrus	36	−24;−32;−16	13	2.56
Parahippocampal gyrus	30	30;−60; 8	139	2.50
Insula	–	−38;−42; 20	42	2.71
Putamen	–	26; 12;−12	22	2.79

**Table 3.4:** Main foci of activation for second person sentences in AVI condition contrasted against RW.

**Region**	**Brodman Area (BA)**	**MNI coordinates**	**Cluster size**	**Peak** **(z score)**
Prefrontal cortex	8	26; 8; 36	61	3.49
	9	−24; 26; 24	19	2.40
	10	−16; 48; 18	19	2.61
Premotor cortex	6	2; 0; 66	66	2.25
		−8; 2; 64		2.12
Inferior frontal gyrus	45	−30; 30; 0	35	2.46
Superior temporal gyrus	22	46;−6;−8	10	2.42
	38	44; 10;−12	30	3.02
Anterior cingulate cortex	24	−8; 30;−2	61	2.47
	32	−12; 22; 22	39	3.16
Posterior cingulate	23	−6;−34; 24	71	2.48
Precuneus	7	−8;−68;40	188	3.37
Caudate	–	16;−34; 22	32	2.97
Cerebellum	–	−6;−46;−36	16	2.27

### Correlations – Questionnaires With fNIRS and fMRI Data

For first person sentences the correlation analysis showed a significant negative relationship between the amplitudes of the precentral gyrus and scores in the LSHS (*r* = −0.482, R^2^ = 0.232, *p* = 0.031), SPQ-G (*r* = −0.642, R^2^ = 0.412, *p* < 0.05) and O-LIFE (*r* = −0.511, R^2^ = 0.261, *p* = 0.021) in the INS condition. For the AVI condition a significant negative relationship between the premotor cortex and scores in the LSHS (*r* = −0.485, R^2^ = 0.235, *p* = 0.030), SPQ-G (*r* = −0.480, R^2^ = 0.230, *p* = 0.032) and O-LIFE (*r* = −0.448, R^2^ = 0.20, *p* = 0.048), between the precentral gyrus and SPQ-G (*r* = −0.595, R^2^ = 0.354, *p* = 0.006) and a significant positive correlation between the amplitudes in the SMG and scores in the VISQ (*r* = 0.561, *p* = 0.012) were evident. No other significant correlations between amplitudes of other ROIs and the questionnaires' scores were found.

For second person sentences, no significant correlations were evident.

The correlation analysis with the fMRI data showed no significant relationship between the amplitudes in the regions of activation and the scores of the questionnaires. In comparison to the fNIRS results the following coefficients of determination were calculated (only the congruent brain regions for fNIRS and fMRI are included): for first person sentences in the AVI condition between the premotor cortex and LSHS (*r* = 0.023; R^2^ = 0.0, *p* = 0.924), SPQ-G (*r* = 0.110; R^2^ = 0.012, *p* = 0.645) and O-LIFE (*r* = −0.073; R^2^ = 0.761). So overall, the comparison of the coefficients of determination (R^2^) showed higher values for fNIRS.

## Discussion

In this study, we utilized an inner speech paradigm during a simultaneous fNIRS-fMRI measurement. The aim was to replicate the findings of neural correlates of inner speech and auditory verbal imagery in healthy subjects, reported in earlier studies. Additionally, our goal was to provide the first validation of the paradigm for fNIRS measurements, in order to investigate neural correlates of these mechanisms in patients with schizophrenia in a subsequent fNIRS study, and potentially link them to AVH. In the paradigm, first and second person sentences had to be constructed and imagined. Study results show that inner speech is normally experienced in first person (30) and AVH usually in second or third (5). As inner speech is claimed to be the primary material of AVHs [e.g., (17–19)], we used these sentence constructions to approach the different manifestations.

All subjects reported that they were able to perform the task. They rated the generation of first person sentences as more feasible and imagining another, alien voice as more difficult. These ratings are consistent with the interpretation that the imaging of an alien voice recruits higher-level brain processes to alter the content from our normal default and could indicate that imagining an alien voice needs more inspection. We investigated a general activation pattern associated with the paradigm, contrasting the different conditions against the baseline. The RW condition was additionally included to subtract the activations associated with reading and processing the presented adjectives in order to specifically investigate the neural correlates of inner speech and different monitoring processes. As predicted, the fNIRS results showed that all conditions elicited activations in the IFG, confirming the hypothesis of its involvement. However, after subtraction of the RW condition, no significant activations in this region were detected, so that the involvement of IFG was not greater during inner speech and monitoring processes than during reading and processing the adjectives. With the existing data, we cannot fully account for this result, but can assume that the RW condition already activated the IFG to a great extent. We hypothesized that all AVI conditions would be associated with activations reported in earlier studies to be involved in monitoring processes, such as the left IFG, bilateral STG, MTG, premotor cortex, SMA, left precentral and postcentral gyri, inferior and posterior parietal lobule. The results show that when contrasted against RW, first person AVI did not elicit any significant activation, while second person AVI showed activation only in the SMG. Therefore, the second hypothesis was only partially confirmed. The third hypothesis, stating that second person INS would involve more active brain areas than first person INS, was confirmed; the same was true for the fourth hypothesis that second person AVI would be associated with more activated regions than first person AVI.

Second person INS elicited activations in Wernicke, premotor area, supramarginal and angular gyrus, which are associated with monitoring processes. The STG is playing an important role in speech perception as well as its phonological and semantic processing [e.g., (44, 45)]. The premotor cortex is playing a role in control of behavior, e.g., planning a movement, and is activated during overt speech [e.g., (44, 46)]. The SMA is claimed to be involved in the initiation of internally generated movement (47) as well as the initiation of articulation (48) and awareness of willed action (49). The involvement of this area is important to identify the self-generated speech as self-generated. The finding, that the SMA was not active during INS or AVI, but the premotor cortex, might be due to the anatomical sensitivity of fNIRS. Because of the lying position of the participants, the upper channels were partly elevated from the head surface, which led to noisier channels covering the parietal lobe.

We expected overall more active brain areas involved in the imagery processes (AVI conditions) compared to INS conditions. The results show, however, that there were no significant differences in activation patterns between INS and AVI in the same sentence form and that the activations were dependent on the construction of the sentences. Second person sentences elicited more active brain areas than first person sentences in both conditions. Furthermore, the second person INS condition elicited more active brain regions compared to the second person AVI condition, showing activations in Wernicke's area, premotor cortex, SMG and angular gyrus when contrasted against RW. This finding has several possible explanations. According to Hurlburt et al. (30) we generate inner speech mainly in first person sentences; still, we did not inquire the form of normally used inner speech. The deployed paradigm did not account for participants who might be used talking to themselves in second person sentences. Hence, we cannot assess to which form the participants were accustomed nor which form might have needed more inspection, involving more brain areas. Imagining the more unfamiliar sentence construction might have been a form of imagery already, independent of whether it was the own voice or an alien voice which had to be imagined. So an explanation for the more active brain regions associated with second person sentences in comparison to first person sentences in both conditions might be that these sentence constructions might have been more unfamiliar and thus needed more inspection, constituting a form of imagery in and of itself. The finding that second person INS elicited activations in brain areas which are associated with imagery could indicate that a form of imagery might have taken place while generating second person sentences in one's own voice. In the second person AVI condition, only the SMG was activated. This finding could indicate that imagining another, alien voice in second person sentences might have been too difficult to perform. The participants rated the imagining of an alien voice as more challenging, but we did not separate between the sentence forms and thus cannot fully account for this finding.

The fMRI results showed greater activation patterns in every condition compared to fNIRS. In accordance with fNIRS results, no activations could be seen in the IFG after contrasting against the RW condition, except in the second person AVI condition. Consistent with the fNIRS results, the fMRI results show greater activation patterns associated with second person sentences in comparison to first person sentences. Overall, the results show congruent activations in fMRI and fNIRS in key brain areas hypothesized to be involved in monitoring processes, such as the STG, premotor cortex, and posterior parietal lobule. This is in line with findings showing that combined fNIRS and fMRI measurements exhibit congruent activations in brain areas most associated with the paradigm (compare) (50). Furthermore, fMRI results showed activations in other regions associated with monitoring processes which cannot be measured by means of fNIRS (e.g., anterior cingulate cortex, parahippocampal gyrus).

To investigate the relationship between the predisposition to acoustic hallucinations and schizotypal traits and the activation pattern associated with different monitoring processes, we correlated the scores in the questionnaires with the activations in the ROIs for the INS and AVI conditions. For the fNIRS data the analyses showed mainly negative correlations in the precentral gyrus and premotor cortex and scores in the LSHS, SPQ-G and O-LIFE for first person sentences (for both, INS and AVI), indicating that higher scores in the schizotypal questionnaires are associated with lower activation in the key areas hypothesized to be involved in monitoring processes. This finding can be interpreted in line with study results showing that patients with schizophrenia exhibit deficient activation associated with monitoring processes [e.g., (22, 24)]. That the negative relationship was evident for first person sentences and not second person sentences, despite overall greater activation associated with second person sentences, can be explained in accordance to the finding of Ehlis et al. (51) showing that greater variance elicits higher correlation scores. As the correlation analysis for the fMRI data and the questionnaires' scores revealed no significant results, we can conclude that fNIRS explained more variance (in the specific case of this study) in the (sub) clinical scales.

The activated areas during inner speech and imagery conditions were similar to those identified as neural correlates of AVHs, indicating a link between these mechanisms. The finding that the auditory cortex was not active in the different conditions indicates that the imagining of one's own and another voice was identified as self-produced speech. In a subsequent study, inner speech and imagery should be investigated in patients with schizophrenia, deploying a similar paradigm to explore their linkage to AVHs.

The study has some limiting factors: As no behavioral performance could be measured, due to the lack of overt outcome, we had little control over whether the participants were executing the task and whether they were able to generate inner speech and imagining according to the instructions. We sought to control the participants' performance by running a training task prior to the experimental task and by ratings of their subjective performance. As the paradigm was a solely mental task, we did not expect a great power. Furthermore, as we did not inquire the form of the normally used inner speech, we cannot conclude which sentence form might have deployed higher demands on the monitoring system and involved more active brain areas and thus cannot provide a sufficient explanation for the discrepancy compared to earlier study results.

There are also some limiting factors associated with the method of fNIRS: because of the supine position of the participants, the upper optodes of the fNIRS probe-set were partly elevated from the head surface, leading to noisier channels, especially in the parietal lobe. Furthermore, biting artifacts, which were controlled for by applying different pre-processing steps, could still have led to discrepancies between both methods in activation in the temporal lobe (e.g., fNIRS results showed significant activations in the MTG, but not the fMRI results; for further information) (52).

### Conclusions

Overall, we were able to replicate findings showing similar brain areas activated during inner speech and monitoring processes to those from earlier studies. However, our results indicate that the activations were dependent more on the sentence form and less on the imaging condition, showing more active brain areas associated with second person sentences. The previous studies did not investigate inner speech in second person sentences and only Shergill et al. (24) investigated imagery in first person sentences. The operationalization of imagery is insufficiently clarified indicating that the concept needs further investigation. Integration of the sentence construction into the model of inner speech and deficient monitoring processes as the basis for the formation of AVHs should be considered in further studies.

As both methods showed similar results, especially in key regions claimed to be associated with monitoring processes, the paradigm seems to be applicable using fNIRS alone. In a subsequent fNIRS study, the paradigm will be deployed with patients with schizophrenia experiencing AVHs.

## Data Availability Statement

The raw data supporting the conclusions of this article will be made available by the authors, without undue reservation.

## Ethics Statement

The studies involving human participants were reviewed and approved by Ethics Committee of the Medical Faculty of the University and the University Hospital of Tübingen. The patients/participants provided their written informed consent to participate in this study.

## Author Contributions

HS: conceptualization, methodology, software, analysis, investigation, and writing—original draft preparation. JH: software, analysis, and writing—reviewing and editing. TD: software, investigation, and writing—reviewing and editing. FH: methodology, software, and analysis. AF: supervision. A-CE: conceptualization and supervision. All authors contributed to the article and approved the submitted version.

## Funding

This research did not receive any specific grant from funding agencies in the public, commercial, or not-for-profit sectors. A-CE was partly supported by the IZKF Tübingen (Junior Research Group 2115-0-0). TD was partly supported by the LEAD Graduate School & Research Network (GSC1028), which is funded within the framework of Excellence Initiative of the German federal and state governments.

## Conflict of Interest

The authors declare that the research was conducted in the absence of any commercial or financial relationships that could be construed as a potential conflict of interest.

## Publisher's Note

All claims expressed in this article are solely those of the authors and do not necessarily represent those of their affiliated organizations, or those of the publisher, the editors and the reviewers. Any product that may be evaluated in this article, or claim that may be made by its manufacturer, is not guaranteed or endorsed by the publisher.
